# Exploring the multi-targeting phytoestrogen potential of Calycosin for cancer treatment: A review

**DOI:** 10.1097/MD.0000000000038023

**Published:** 2024-05-03

**Authors:** Fangbing Ren, Yanhui Ma, Kexin Zhang, Youhong Luo, Ruiyan Pan, Jingwen Zhang, Chengxia Kan, Ningning Hou, Fang Han, Xiaodong Sun

**Affiliations:** a Department of Endocrinology and Metabolism, Affiliated Hospital of Weifang Medical University, Weifang, China; b Clinical Research Center, Affiliated Hospital of Weifang Medical University, Weifang, China; c Department of Pathology, Affiliated Hospital of Weifang Medical University, Weifang, China; d School of Pharmacy, Weifang Medical University, Weifang, China.

**Keywords:** antitumor, Calycosin, cancer, mechanism, tumor

## Abstract

Cancer remains a significant challenge in the field of oncology, with the search for novel and effective treatments ongoing. Calycosin (CA), a phytoestrogen derived from traditional Chinese medicine, has garnered attention as a promising candidate. With its high targeting and low toxicity profile, CA has demonstrated medicinal potential across various diseases, including cancers, inflammation, and cardiovascular disease. Studies have revealed that CA possesses inhibitory effects against a diverse array of cancers. The underlying mechanism of action involves a reduction in tumor cell proliferation, induction of tumor cell apoptosis, and suppression of tumor cell migration and invasion. Furthermore, CA has been shown to enhance the efficacy of certain chemotherapeutic drugs, making it a potential component in treating malignant tumors. Given its high efficacy, low toxicity, and multi-targeting characteristics, CA holds considerable promise as a therapeutic agent for cancer treatment. The objective of this review is to present a synthesis of the current understanding of the antitumor mechanism of CA and its research progress.

## 1. Introduction

Cancer has become one of the diseases with the highest mortality rate worldwide.^[1]^ Cancer treatment options include surgical intervention, radiotherapy, chemotherapy, and targeted therapy. However, cancer research focuses on developing therapeutic strategies with low toxicity and high specificity.^[2]^ Herbal medicines hold immense promise with their advantages of safety, low cost, and minimal side effects. It is crucial to purify and extract the effective components of these medicines for further study to minimize the impact of impurities on disease. One such valuable resource is *Calycosin* (CA), a bioactive phytoestrogen isoflavone derived from traditional Chinese medicinal plants such as Radix Hedysarum and Radix Astragali. CA has been demonstrated to possess a range of biological activities, including anti-inflammatory, antioxidant, anti-osteoporosis, and anti-diabetic effects.^[3,4]^ Additionally, it has been shown to offer protective effects on the heart, blood vessels, nerves, liver, kidney and against cancer.^[5–9]^ Notably, several studies have highlighted the potential of CA as an anti-metastatic agent in various tumors, promoting apoptosis in cancer cells while exhibiting low toxicity to normal cells.^[10]^ Despite these promising findings, the specific mechanism of action and its impact on different cancers remains poorly understood. In this review, we aim to provide a comprehensive overview of the biological activity and underlying mechanisms of CA, highlighting its inhibitory effects on multiple cancers. We discuss the structure, dietary sources, pharmacokinetics, and pharmacology of CA, emphasizing its diverse mechanisms of action in cancer prevention and treatment. Furthermore, we delve into the specific anticancer effects of CA in various types of cancers. The review utilized a search strategy with CA-related keywords alongside cancer, across PubMed, Web of Science, and Embase. Duplicate records were removed, focusing solely on studies directly relevant to the role of CA in cancer and therapeutic targeting.

## 2. Structure, source, pharmacokinetics and pharmacology of CA

### 2.1. Structure and source of CA

CA (7,3′-dihydroxy-4′-methoxy isoflavone, C16H12O5) is one of the current research hotspots in herbal medicine research. CA is a bioactive phytoestrogen isoflavone extracted from traditional Chinese medicinal plants such as Astragalus membranaceus,^[11]^ Hedysarum Polybotry,^[12]^ Glycyrrhiza glabra,^[13]^ Spatholobi Caulis^[14]^ (Table 1), with the highest content of CA found in Astragalus membranaceus.^[15]^ In its pure form, CA is a white needle-like crystal that displays poor solubility in water and various organic solvents, including ethanol, methanol, and chloroform. It also shows poor solubility in acetone and dichloromethane.

**Table 1:** Contents of Calycosin in different plants.

Plant origin	Concentration	Ref.
Astragalus membranaceus	35.8–98.5 μg/g	^[11]^
Hedysarum Polybotrys	1.02–4.03 μg/g	^[12]^
Glycyrrhiza glabra	Unknown	^[13]^
Spatholobi Caulis	Unknown	^[14]^

### 2.2. Pharmacokinetics of CA

**Figure 1.**
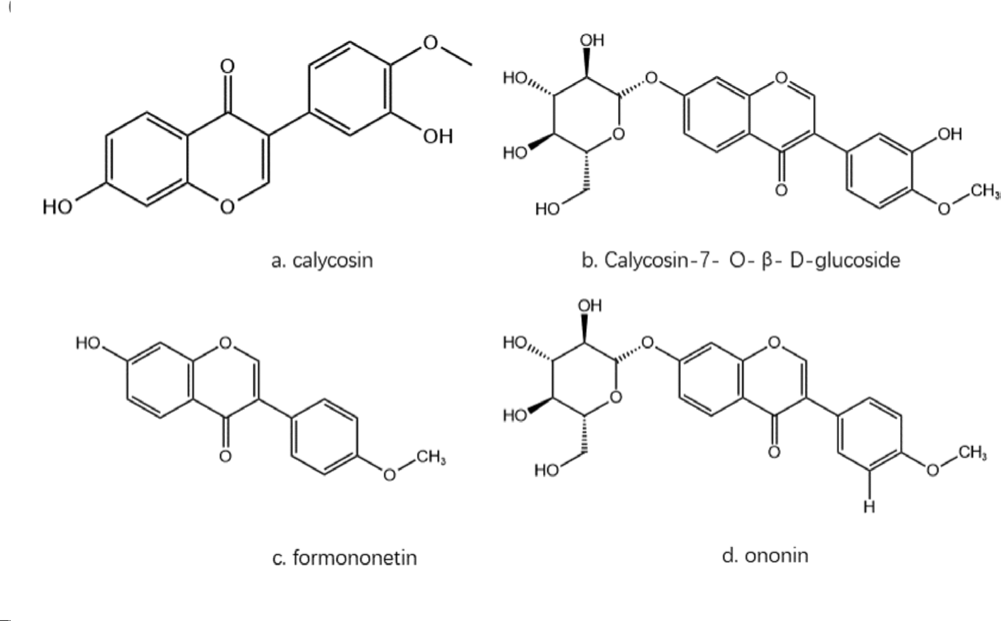
Chemical structures of the 4 flavonoids.

The pharmacokinetics of CA have been thoroughly studied and it was found to display the fastest absorption and elimination among 4 isoflavones (CA, CA-7-O-β-D-glucoside, ononin and formononetin) (Fig. 1) after oral administration of Astragalus membranaceus extract solution.^[16]^ The primary sites of CA metabolism are the intestine and liver, with the highest absorption and permeability observed in the colonic segment of the intestine. CA is primarily metabolized in the liver as flavonoid sugars, while those absorbed in the intestine are secreted into the intestinal lumen before reaching the liver, leading to its low bioavailability. The rapid absorption and elimination of CA highlight its potential as a promising therapeutic agent in treating various diseases.^[17]^

Studies have suggested a potential correlation between blood calcium levels and CA absorption. Zeng et al^[18]^ conducted a study where rats were administered Astragalus decoction supplemented with varying doses of calcium and found that the peak concentration, time to reach peak concentration, and overall exposure of CA increased in proportion to the dose. Furthermore, it is believed that CA is transported into the intestine by calcium transporters in the intestinal epithelial cells and can circulate between the epithelial cells and the gastrointestinal tract. Upon oral administration of Astragalus extract to rats, plasma CA concentrations were consistently below the minimum quantifiable limit. This phenomenon is hypothesized to be due to the rapid glucuronidation of CA in the intestinal epithelial cells, resulting in its circulation in the bloodstream at lower concentrations.^[19]^

In subcellular fractions derived from the human liver, CA can be metabolized into 3′-glucuronide (G1) and 7-glucuronide (G2). The affinity of CA to the former is higher due to a higher affinity of the human liver microsomes to G1. These findings provide insight into the pharmacokinetics of CA and highlight the need for further studies to fully understand its absorption and elimination mechanisms.^[20,21]^

### 2.3. Pharmacology of CA

CA has been extensively studied for its multiple pharmacological properties, including anti-inflammatory, antioxidant, anti-osteoporosis, and anti-diabetic effects. It has been demonstrated to protect the heart, blood vessels, nerves, and liver from various forms of injury and disease.^[6,22]^ For example, CA and its derivatives have been shown to protect cardiac muscle cells against damage caused by cardiac enzymes. This is believed to occur through activation of the transient receptor potential paradigm 6 pathway and other protective proteins.^[23]^ In addition, CA supplementation has been shown to mitigate liver injury induced by a high-fat diet, highlighting its potential as a therapeutic strategy for preventing metabolic disorders.^[5,24]^ Additionally, CA has demonstrated potential as an anti-metastatic agent in numerous studies, displaying the ability to suppress cancer cell growth, increase apoptosis in cancer cells, and exhibit minimal toxicity to normal cells.^[3,25]^ However, it is important to note that CA has also been shown to induce breast cancer metastasis when administered at high doses in conjunction with paclitaxel.^[26]^ This is due to its angiogenic and vascular permeability-promoting effects. Therefore, it crucial to highlight the need for a comprehensive understanding of the multiple roles of CA in different tumor tissues.

## 3. CA in various cancers

**Figure 2.**
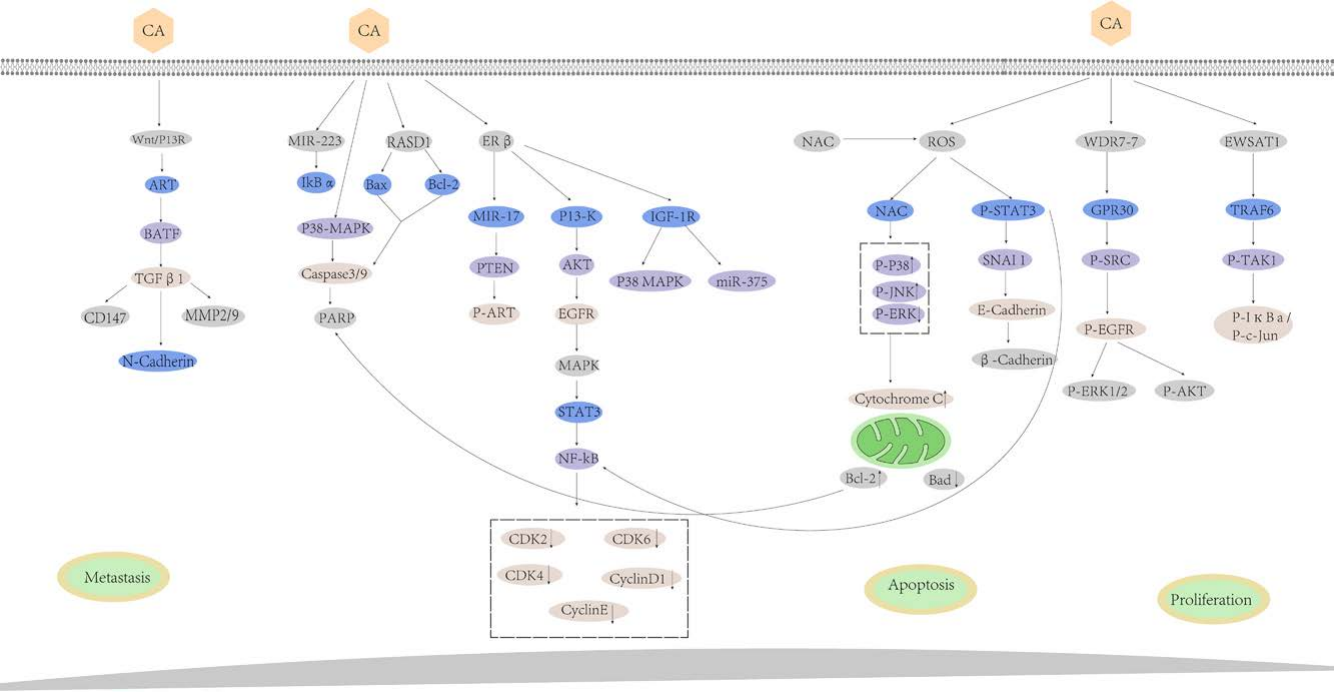
Antitumor mechanism of Calycosin. Akt = protein kinase B, Bad = Bcl-xL/Bcl-2asociated death promoter, Bcl-2 = B-cell lymphoma-2, BATF = The basic leucine zipper ATF-like transcription factor, Caspase-3 = cysteinyl aspartate specific proteinase-3, CDK2 = cyclin-dependent kinase 2, c-Jun = c-N-terminal kinase, EGFR = epidermal growth factor receptor, ERK = extracellular regulated protein kinases, ERβ = estrogen receptor β, EWSAT1 = Ewing sarcoma-associated transcript 1, GPR30 = G-protein coupled estrogen receptor 30, IGF-1R = insulin-like growth factor 1, IκBα = inhibitor of NF-κB α, JNK = c-Jun N-terminal kinase, MMP-9 = matrix metalloproteinase-9, miR-375 = microRNA-375, MAPK = mitogen-activated protein kinase, NF-κB = nuclear factor kappa B, NAC = n-acetyl-l-cysteine, PARP = poly(ADP-ribose)polymerase, PTEN = phosphatase and tensin homolog deleted on chromosome 10, PI3K = phosphoi nositide 3-kinase, RASD1 = RAS dexamethasone-induced 1, ROS = reactive oxygen species, STAT3 = signal transducer and activator of transcription 3, TRAF6 = tumor necrosis factor receptor-(TNFR)-associated factor 6, TAK1 = transforming growth factor-β-activated kinase 1, TGF-β-1 = transforming growth factor-β, WDR7-7 = WD repeat-containing protein 7.

Cancer has several hallmark features, including sustained cellular proliferation, programmed cell death (apoptosis) evasion, and genomic instability.^[27]^ The alterations of various signaling pathways and protein activities in cancer cells contribute to the dysregulation of their growth, proliferation, apoptosis, invasion, and metastasis^[28]^ (Fig. 2). Additionally, the estrogen receptor (ER), comprised of 2 subtypes (ERα and ERβ), plays a crucial role in some cancers dependent on estrogen. The balance between the levels of ERα and ERβ affects cellular proliferation, with a higher ratio of ERα to ERβ promoting cell growth and a lower ratio suppressing it.^[29,30]^ Tumor tissues typically express lower levels of ERβ, and some cancers related to estrogen dependence may act through ERβ, such as colorectal, breast, and renal cell cancers.^[31,32]^ These findings suggest that ERβ may have a tumor-suppressive role and maybe a potential target for cancer therapy. Notably, CA has been shown to exert anticancer effects in a wide range of tumors while acting mainly by inducing apoptosis and reducing the proliferation of cancer cells. Moreover, CA inhibits osteosarcoma and breast and colorectal cancers by regulating ERβ.^[33–35]^ These effects are tumor-specific, and the mechanisms may vary between different types of tumors. Table 2 highlights the specific effects of CA in pan-cancer.

**Table 2:** The anticancer mechanism and effects of Calycosin.

Cell lines/model	Cancer	Mechanism	Effect	Concentration	Ref.
MCF-7 cell	Breast cancer	Upregulates RASD1	Through the mitochondrial apoptotic pathway	20, 50, 100 μM	^[36]^
MCF-7 cell, T47D cell	Breast cancer	Reduces Foxp3, VEGF and MMP-9	Inhibition of cell migration and invasion	50, 100, 150 μM	^[37]^
MDA-MB-231 cell	Breast cancer	Inhibits Rab27B, β-linked protein and VEGF	Inhibition of cell migration and invasion	150 μM	^[38]^
MCF-7, T47D, SKBR3, MDA-MB-468, MDA-MB-231, MCF10A cells; MCF-7 and SKBR3 nude mice	Breast cancer	Inactivates MAPK and PI3K/Akt pathways by downregulation of SRC and EGFR via the WDR7-7-GPR30 pathway	Inhibition of cell proliferation	1–32 μM; 55 mg/kg/d	^[10]^
MCF-7, T47D, MDA-231 and MDA-435 cells	Breast cancer	Upregulates ERβ isoforms, down-regulates IGF-1R, regulates MAPK and PI3K/Akt pathways	Inhibition of cell growth and induction of apoptosis	25–100 μM	^[31]^
MCF-7 cell	Breast cancer	Inactivates HOTAIR/p-Akt signaling pathway	Inhibition of proliferation and induction of apoptosis	80 μM	^[39]^
MCF-7, T47D cell, MDA-231 and MDA-435 cells	Breast cancer	ERβ-mediated regulation of IGF-1R signaling pathways and miR-375 expression	Inhibition of proliferation and induction of apoptosis	25–100 μM	^[34]^
MCF-7 cell, T47D cell, T47D nude mice	Breast cancer	Increases E-cadherin and decreases N-cadherin, Vimentin, CD147, MMP-2, and MMP-9 levels through downregulation of BATF and TGFβ1	Inhibition of EMT	200, 400 μM	^[40]^
AGS cell	Gastric carcinoma	Upregulates MAPK/STAT3/ NF-κB	G0/G1 cell cycle arrest and inhibition of cell growth and migration	47 μM	^[41]^
Human gastric cell lines SGC7901, BGC823, and NCIN87	Gastric carcinoma	Inhibits Akt phosphorylation, down-regulates MMP9 and MMP2	Inhibition of growth, migration and invasion of cells	10 µg/mL	^[42]^
SW480, LoVo and HeLa cells, female mice (4–5 wk)	Colorectal cancer	Regulates ER β/MiR-95 and IGF-1 R, PI3K/Akt signaling pathways	Inhibition of cell proliferation and induction cell apoptosis	10–80 μM; 10, 20, 40 mg/kg	^[32]^
HCT-116 cell	Colorectal cancer	Increases ERβ, thereby reducing miR-17 and increasing PTEN	Inhibition of cell proliferation, apoptosis and migration	100 μM	^[33]^
HCT-116, LoVo human CRC cells	Colorectal cancer	Upregulates BATF2	Inhibition of cell migration and induction of cell apoptosis	50, 100, 150 μM	^[43]^
HT29 cell	Colorectal cancer	Activates SIRT1, triggersAMPK to inhibit the Akt/mTOR axis	Promotes cell apoptosis and inhibits their invasion	50, 80 μM	^[44]^
HepG2 cell	Hepatoma	Promotes MAPK, STAT3 and NF-κB pathways	Inhibition of cell migration and induction of cell apoptosis	100 μM	^[45]^
Human PDAC cell lines PANC1 and MIA PaCa-2, BALB/c mice	Pancreatic cancer	Induces p21Waf1/Cip1-induced cell cycle arrest and caspase-dependent apoptosis	Inhibition of cell growth and migration	25–200 μM; 15 or 30 mg/kg	^[46]^
143B cell and human osteoblast cell line (hFOB1.19)	Osteosarcoma	P38-MAPK regulation of mitochondrial-dependent intrinsic apoptotic pathways	Inhibition of apoptosis	0–160 µg/mL	^[47]^
143B cell, 143B tumor-bearing nude mice	Osteosarcoma	Inhibits miR-223-IκBα signaling pathway	Inhibition of apoptosis	60, 120, 180 mM 30, 60, 120 mg/kg/d	^[48]^
MG-63 and U2-OS cells, MG-63 tumor-bearing nude mice	Osteosarcoma	Promotes PI3K/AKT/mTOR pathway	Inhibition of apoptosis	25, 50, 100 μM; 2, 4, 8 mg/kg	^[22]^
MG-63 and human fetal osteoblast cells (hFOB1.19)	Osteosarcoma	ERβ-dependent inhibition of PI3K/Akt pathways	Inhibition of proliferation and induction of apoptosis	100 µM	^[35]^
143B and human osteoblast cells (hFOB1.19), 143B tumor-bearing nude mice	Osteosarcoma	Upregulates intracellular Apaf-1 and cleaved Caspase-3 protein levels; down-regulates Bcl-2 protein	Induction of apoptosis	20, 40, 80, 160 μg/mL; 2, 4, 8 mg/kg	^[49]^
143B cell and 143B tumor-bearing nude mice	Osteosarcoma	Inactivates neoplastic IκBα/ECT2 pathway	Induction of cell apoptosis and Inhibition of cell migration	60, 120, 180 μM; 30, 60, 120 mg/kg	^[50]^
U87 and U251 cells, U87 tumor-bearing nude mice	Glioblastomas	Inhibits TGF-β, Snail and Vimentin	Inhibition of cell migration and invasion	100, 200 μM; 7.5 mg/kg	^[51]^
HEK293T, U251 and U81 cells	Glioblastomas	Targets on c-Met and exerts an antitumor role via MMP9 and Akt	Inhibition of cell proliferation, invasion and induction of cell apoptosis	800 μM	^[52]^
SKOV3 cell	Ovarian tumor	Activates caspases and Bcl-2 family proteins	Inhibition of growth and induction of apoptosis	25, 50, 100 μM	^[53]^
NPC cell lines; CNE1 cell mice model	Nasopharyngeal carcinoma	Regulates EWSAT1 and its downstream pathway	Inhibition of cell proliferation	50 μM; 60 mg/kg	^[54]^
CL1-0 cell, CL1-0 GEMR cell and human fetal lung fibroblast	Lung cancer	Increases LDOC1 and decreases GNL3L/NF-κB	Inhibition of cell proliferation	50, 100, 150, 200 μM	^[55]^
A549 cell	Lung cancer	Induces PKC-α/ERK1/2 repression, upregulates E-cad expression, and inhibits MMP-2, MMP-9, and integrin β1	Inhibition of proliferation, adhesion, migration and invasion of TPA-induced A549 cells	10–90 µM	^[56]^
B-CPAP cell	Thyroid cancer	Via SESN2/AMPK/mTOR pathway	Restrain cell proliferation, migration and invasion	100 μM	^[7]^
SiHa, CaSki, C-33A and HeLa cells	Cervical cancer	Upregulates tumor suppressor miR-375	Inhibit proliferation, induce apoptosis and prevent invasion	30, 40, 50 μM	^[57]^
K562 cell	Erythroleukemia cells	Arrests in G0/G1 phase, decreases cyclin D1	Inhibition of cell proliferation	20–400 μg/mL	^[58]^

Akt = protein kinase B, AMPK = AMP-activated protein kinase, BATF = The basic leucine zipper ATF-like transcription factor, Bcl-2 = B-cell lymphoma-2, EGFR = epidermal growth factor receptor, ECT2 = epithelial cell transforming sequence 2, EWSAT1 = Ewing sarcoma-associated transcript 1, ERβ = estrogen receptor β, Foxp3 = fork head box P3, GPR30 = G-protein coupled estrogen receptor 30, GNL3L = guanine nucleotide binding protein-like 3-like, HOTAIR = the HOX transcript antisense RNA, LDOC1 = leucine zipper downregulated in cancer 1 gene, IGF-1R = insulin-like growth factor 1, IκBα = inhibitor of NF-κB α, MMP-9 = matrix metalloproteinase-9, MMP-2 = matrix metalloproteinase-2, MAPK = mitogen-activated protein kinase, miR-375 = microRNA-375, NPC = nasopharyngeal carcinoma, NF-κB = nuclear factor kappa B, PTEN = phosphatase and tensin homolog deleted on chromosome 10, RASD1 = RAS dexamethasone-induced 1, ROS = reactive oxygen species, STAT3 = signal transducer and activator of transcription 3, SIRT1 = Sirtuin1, TGFβ1 = transforming growth factor-β, VEGF = vascular endothelial growth factor, WDR7-7 = WD repeat-containing protein 7.

### 3.1. Breast cancer

Breast cancer (BC) is a leading cause of cancer mortality among women worldwide. The current standard of care for BC treatment involves a multi-disciplinary approach, including surgery, radiation therapy, adjuvant and neoadjuvant therapies.^[59,60]^ Despite significant progress in the treatment of BC in recent decades, patients with advanced stages of the disease continue to face poor prognoses due to drug resistance and high rates of disease recurrence.^[61,62]^ Recently, CA has emerged as a promising treatment option for BC.^[40]^ Studies have shown that CA can effectively suppress the proliferation of BC cells, induce apoptosis, and control the migration and invasion of BC cells.^[37]^

BC is a complex disease with diverse subtypes, including ER-positive and ER-negative BC. ER-positive BC is dependent on the presence of estrogen, and its development and progression have been linked to increased levels of circulating estrogen.^[63]^ The interaction between estrogen and ERs can drive tumor growth, making modulation of ERs a potential therapeutic target for ER-positive BC. Interestingly, CA significantly impacts ER-positive BC cells (such as MCF-7 and T47D cells) by inhibiting their proliferation. Conversely, CA has fewer effects on ER-negative BC cells (such as MDA-MB-231 cells).^[31,38]^ These findings suggest that CA could be a promising treatment option for ER-positive BC by modulating the activity of ERs.

The insulin-like growth factor-1 receptor (IGF-1R) regulates cell proliferation, differentiation, death, transformation, and other vital physiological processes via cell signaling.^[64]^ On this basis, Chen et al^[31]^ demonstrated that CA could inhibit the proliferation of ER-positive BC cells through ERβ-mediated regulation of IGF-1R-mediated mitogen-activated protein kinase (MAPK) and PI3K/Akt pathways. CA can also act through the ERβ-mediated IGF-1R signaling pathway and miR-375 expression in ER-positive BC cells.^[34]^ Additionally, the study showed that combining CA with genistein could impact HOTAIR expression through the PI3K/Akt pathway, leading to an increase in apoptosis of BC cells.^[39]^

In addition, CA inhibits MCF-7 cell and T47D cell invasion and metastasis in a dose-dependent manner by inhibiting Forkhead box P3 (Foxp3) and reducing vascular endothelial growth factor and matrix metalloproteinase-9 (MMP-9).^[37]^ The study by Tian et al^[36]^ supports the above notion that the *Ras-MAPK* signaling pathway is activated in response to high concentrations (50 μM) of CA induced MCF-7 and triggers apoptosis through the mitochondrial apoptotic pathway. These results showed that CA upregulated *RASD1* expression and the mitochondrial apoptotic pathway of B-cell lymphoma-2 (*Bcl-2*) and *Bax* protein expression, triggering apoptosis and inhibiting BC cell proliferation. *Basic albino zipper ATF-like transcription factor (BATF*) is a basic albino zipper nuclear protein that belongs to the activating protein-1*/ATF* protein superfamily.^[65]^ Zhang et al^[40]^ discovered that CA could inhibit the migration and invasion of BC cells by reducing *BATF* expression and inhibiting the *BATF/TGFβ1* pathway.

Several studies have also confirmed that CA has an inhibitory effect on some ER-negative BC cells. The secretory *Rab27B* small GTPase is known to promote the aggressive growth of ER-positive BC cells.^[66]^ However, research by Wu et al^[38]^ has indicated that in the ER-negative BC cell line *MDA-MB-231, Rab27B* expression is positively correlated with cancer cell aggressiveness. Treatment of *MDA-MB-231* cells with CA was found to reduce cell migration and invasion by inhibiting *Rab27B-dependent* signaling. Furthermore, a study by Tian et al^[10]^ showed that CA can hinder the growth of both ER-positive and ER-negative BC cells via *WDR7-7-GPR30* signaling. Overall, these studies suggest that CA may be a promising therapeutic option for treating BC by modulating multiple signaling pathways involved in cell proliferation, migration, and apoptosis.

### 3.2. Gastric cancer

Gastric cancer (GC) is a severe disease with a low survival rate and a median survival time of less than 1 year for metastatic cases.^[67,68]^ One of the major causes of this cancer is the bacterium Helicobacter pylori, which can lead to precancerous lesions such as atrophic gastritis and intestinal epithelial metaplasia.^[69]^ Chemotherapy is the primary treatment for GC, but intrinsic or acquired resistance to drugs like cisplatin, 5-fluorouracil, and Adriamycin has diminished their effectiveness.^[70]^

Studies have shown that CA may enhance the inhibition of these chemotherapy drugs on GC cells by suppressing the protein *kinase B (Akt*) phosphorylation.^[42]^ Gastric precancerous lesions, including intestinal epithelial chemosis and heterogeneous hyperplasia, are the basis of GC development and are linked to inflammation that promotes malignant cell proliferation and survival, angiogenesis, and metastasis.^[71–73]^ CA has been shown to protect the gastric mucosa from injury in a gastric precancerous lesion mouse model by modulating the integrin *β1*/nuclear factor kappa B (*NF-κB)/DARPP-32* pathway.^[74,75]^

**Figure 3.**
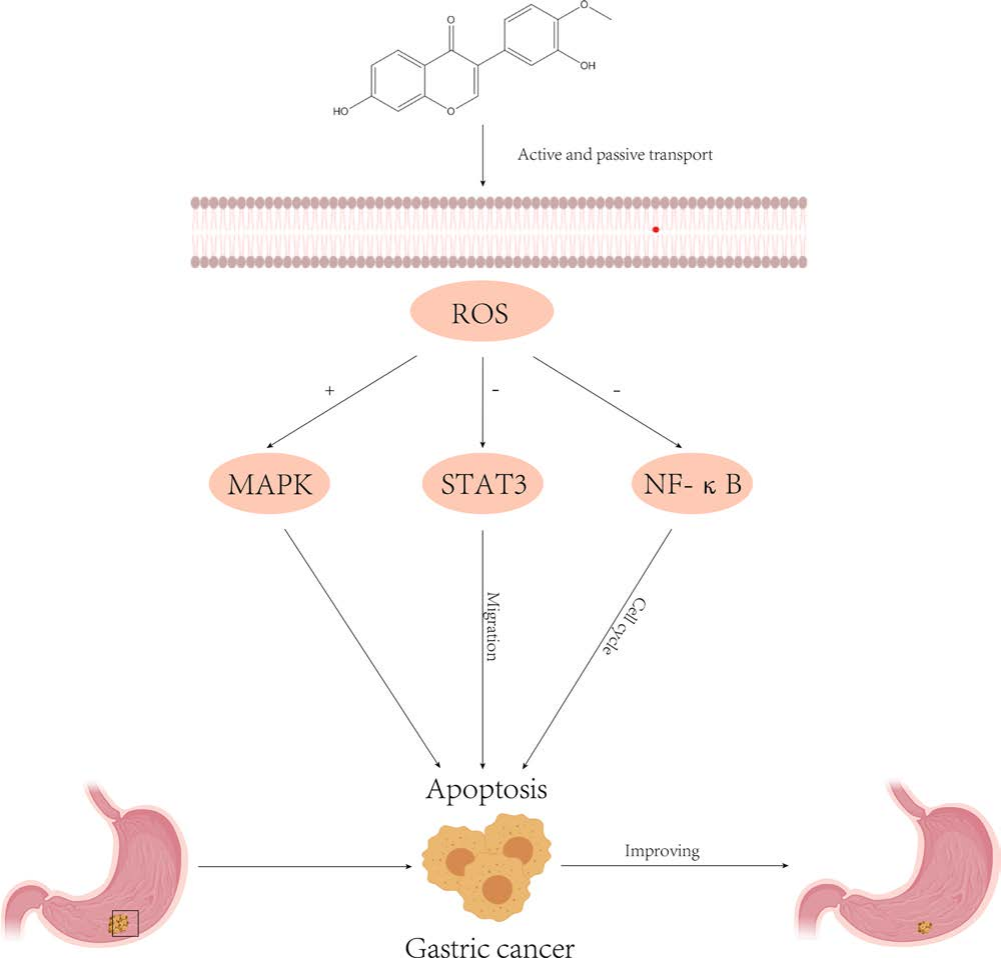
Mechanism of action of Calycosin in gastric cancer. + = Promotion; − = inhibition, MAPK = mitogen-activated protein kinase, NF-κB = nuclear factor kappa B, ROS = reactive oxygen species, STAT3 = signal transducer and activator of transcription 3.

Mitochondria in tumor cells produce more *reactive oxygen species (ROS*) than normal cells, which can promote apoptosis and inhibit cancer cell metastasis through regulation of *MAPK*, signal transducers and activators of signal transducer and activator of transcription 3 (*STAT3*), and *NF-κB* signaling pathways.^[76–78]^ Due to its relatively low polarity, CA can penetrate the lipid bilayer, counteract lipid peroxidation, and scavenge oxidative free radicals.^[79]^ Zhang et al^[41]^ found that CA can increase *ROS* production through the *MAPK/STAT3/NF-κB* pathway, prevent AGS cell (GC cell) development in the G0/G1 phase, inhibit cell migration, and induce apoptosis, thus exhibiting anticancer effects (Fig. 3).

### 3.3. Colorectal cancer

Colorectal cancer is the third most prevalent cancer worldwide, but its incidence is lower in Japan and China, where the consumption of phytoestrogen-rich soy products is more common.^[80,81]^ It has been suggested that increased intake of phytoestrogens may reduce the risk of colorectal cancer, indicating that these compounds may have anticancer properties.^[43]^ Zhao et al^[32]^ demonstrated that CA treatment significantly inhibited tumor growth in mice xenografts. This effect was associated with the downregulation of Akt phosphorylation, IGF-1R, and ERα, via the upregulation of ERβ and *miR-95*. Similarly, another study found that CA could suppress the proliferation and invasion of colorectal cancer cells by increasing the expression of ERβ and *phosphatase and tensin homolog deleted on chromosome 10 (PTEN*) while decreasing *miR-7* expression.^[33]^ Huang et al^[82]^ identified ESR2, ABCG2, BRCA1, ESR1, CYP19A1, and *EGFR* as potential targets for CA treatment in colorectal cancer. CA was also found to activate Sirtuin1 (*SIRT1*), inhibiting autophagy and suppressing the *Akt/mTOR* axis, thereby promoting apoptosis and inhibiting tumor cell invasion in colorectal cancer cells.^[44]^

### 3.4. Hepatocarcinoma

Hepatocellular carcinoma (HCC) is the most prevalent primary liver cancer and the second-leading cause of cancer-related mortality worldwide.^[83]^ During HCC progression, cell cycle defects can occur, and several signaling pathways can be activated, including *MAPK, STAT3*, and *NF-κB*.^[84–86]^ CA has been shown to regulate the cell cycle, inhibit cell migration, and induce mitochondria-dependent cell apoptosis in HCC. Specifically, CA blocked the G1/S and G2/M transitions in the BEL-7402 human HCC cell line by regulating cell cycle checkpoints through significant downregulation of *transcription factor DP-1 (TFDP-1*) and *SKP2* expression.^[45,87,88]^ Another study also demonstrated that CA could induce cellular G0/G1 phase block in HCC by regulating cell cycle proteins.^[45]^ Furthermore, CA induced apoptosis in HepG2 cells through the mitochondrial route, as well as the *ROS*-mediated *MAPK, STAT3,* and *NF-κB* signaling pathways.^[45]^ When combined with *IFN-γ*, CA can exert synergistic effects that enhance the electrostatic force and improve the stability of *IFN-γ* secondary and tertiary structures, thereby improving the anti-liver cancer effect.^[89]^ However, further studies are needed to verify the practical effects of this combination, as current evidence is limited to cellular and molecular levels.

### 3.5. Pancreatic cancer

Pancreatic cancer is a devastating disease with a poor prognosis, with a 5-year survival rate of <10%.^[90]^ Early surgery is currently the only effective treatment, but once the tumor metastasizes, patients will have no effective treatment. Luckily, CA has been shown to inhibit pancreatic cancer growth through *p21*-induced cell cycle arrest and cystine-dependent apoptosis.^[46]^ Additionally, CA increased the *Bax/Bcl-2* ratio, inducing apoptosis through the mitochondrial pathway.^[46,91]^ Nevertheless, it is essential to note that CA plays a “dual role” in pancreatic cancer, as it has been found to trigger epithelial-mesenchymal transition (EMT) and matrix metalloproteinases activation through the *TGF-β-driven Raf/MEK/ERK* pathway, promoting pancreatic cancer metastasis.^[46,92]^ Further experiments are needed to confirm whether CA has an inhibitory effect on pancreatic cancer.

### 3.6. Osteosarcoma

Osteosarcoma is a malignancy that affects adolescents and has limited treatment options due to chemotherapy resistance and metastasis.^[93]^ Estrogen-related endocrine therapy may be effective since sex hormones are linked to osteoporosis.^[94]^ CA has shown promise in limiting osteosarcoma cell growth and metastasis by modulating ER expression and various signaling pathways. First, in the study by Qiu et al, CA could exert antitumor effects through the induction of apoptosis and inhibition of the *miR-223-IκBα* signaling pathway.^[48]^ Tian et al^[35,47]^ showed that CA inhibited osteosarcoma cell growth through *p38-MAPK* pathway and ERβ-dependent regulation of *PI3K/Akt* pathway. Second, when CA acts on ER-positive MG-63 tumor cells, it can achieve antitumor effects via the *PI3K/Akt/mTOR* pathway.^[22]^ Third, CA increased the expression of caspase-3 protein and apoptosis protease-activating factor-1 in osteosarcoma cells while lowering the expression of intracellular *B-cell lymphoma 2 (Bcl2*) protein.^[49]^ Finally, epithelial cell transforming 2, an exchange factor in the Rho band, is a potent metastatic oncoprotein. Osteosarcoma metastasis may be associated with this oncoprotein overexpression.^[95]^ Interestingly, CA inhibited *inhibitor of NF-κB α (IκBα*) activation and epithelial cell transforming 2 overexpression by reducing functional downstream proteins such as *IL-6* and *MMP2* to achieve anti-osteosarcoma metastasis function.^[50]^

### 3.7. Glioblastoma

Glioblastoma is a highly aggressive central nervous system tumor with a poor prognosis.^[96]^ While progress has been made in our understanding of glioblastoma, patients still face a median overall survival of approximately 15 months.^[97]^ Glioblastoma development is linked to EMT induced by *matrix metalloproteinases (MMP*), particularly *MMP-2* and *MMP-9*, which promote tumor aggressiveness by degrading the extracellular matrix and promoting the *EMT* process.^[98–100]^ TGF-β regulates *EMT* and *MMP* activation in various cancer cells.^[101–103]^ In the case of U87 and U251 cells, CA has been shown to inhibit the *EMT* process by downregulating *TGF-β* and suppressing *MMP-2* and *MMP-9*.^[51]^ Additionally, CA was found to target and inhibit *c-Met*, a receptor tyrosine kinase, at high concentrations (≥50 μM), thereby suppressing glioblastoma development through *Akt* and *MMP9*.^[52]^ These findings suggest that CA may have therapeutic potential as a treatment for glioblastoma.

### 3.8. Lung cancer

Lung cancer is the leading cause of cancer-related mortality globally, and smoking is a significant contributing factor. Lung adenocarcinoma is a prevalent type of non-small cell lung cancer. The migration and invasion of cancer cells are closely related to intercellular adhesion, which is facilitated by critical proteins such as *E-cadherin (E-cad*) and *integrin β1*. Cheng et al^[56]^ demonstrated that CA inhibits the metastasis of A549 human lung adenocarcinoma cells by inhibiting the *PKC-α/ERK 1/2* signaling pathway, increasing E-cad expression, and improving adhesion between cancer cells. Guanine nucleotide binding protein-like 3-like (GNL3L), a nucleolar GTP-binding protein, promotes cell proliferation and is overexpressed in various tumors; LDOC1 regulates NF-κB activity, while GNL3L acts as a mediator of the NFκB pathway.^[104,105]^ CA inhibited the proliferation of gemcitabine-resistant lung cancer cells by regulating the LDOC1/GNL3L/NF-κB pathway, thereby offering novel therapeutic possibilities for treating drug-resistant lung cancer.^[55]^

### 3.9. Other cancers

CA has also been found to potentially protect against other cancers, including nasopharyngeal carcinoma (NPC), ovarian cancer, thyroid cancer, cervical cancer, and leukemia. NPC, a prevalent type of tumor in East and Southeast Asia, can be inhibited by CA by regulating ewing sarcoma-associated transcript 1 and its downstream factors (*TRAF6, pTAK1, and p-IкBa/p-c-Jun*), as demonstrated by Kong et al^[54]^ Moreover, CA significantly reduced tumor weight in tumor-bearing nude mice and served to inhibit tumor growth.^[54,106]^ Additionally, Liu et al^[107]^ used bioinformatics and experiments to identify *TP53, MAPK14, CASP8, MAPK3, CASP3, RIPK1, JUN,* and *ESR1* as possible therapeutic targets for NPC treated with CA. Regarding ovarian cancer, Zhou et al^[53]^ showed that CA could upregulate *Bax/Bcl-2* and decrease the expression of *caspase-3* and *caspase-9* in a dose-dependent manner, thus acting as an anti-growth agent against ovarian cancer cells. However, the current studies on CA in ovarian cancer are limited. In the case of thyroid cancer, our group showed that CA could promote apoptosis and autophagy through the *SESN2/AMPK/mTOR* pathway, thereby inhibiting the proliferation and invasion of thyroid cancer r.^[7]^ For cervical cancer, CA inhibited cancer cells and reduced the invasiveness of cervical cancer cells by inducing apoptosis through upregulation of the tumor suppressor *miR-375*, as studied by Zhang et al^[57]^ CA has shown good cytotoxic activity against adult lymphocytic leukemia *CEM-13* cells and human T-cell leukemia MT-4 cells.^[108]^ Additionally, CA blocked cell proliferation in the G0/G1 phase and induced a decrease in cyclin D1 mRNA in human erythroleukemia cells K562, as demonstrated by Zhang et al^[58]^

## 4. Conclusion

In summary, CA is an active herbal extract that has exhibited potent antitumor effects in various cancers. It can also be combined with certain chemotherapy drugs like cisplatin to enhance its antitumor effects. However, the metabolism of CA varies widely among different tumors, drug combinations, and doses, which may be attributed to the metabolizing enzymes of CA. Further, detailed pharmacokinetic studies on the metabolizing enzymes of CA and the effect of CA on the metabolism of other components are required. Although CA has low toxicity, potential adverse reactions or toxicity due to the inhibition of metabolic enzyme activity of endogenous substance metabolism must be considered. Additionally, a growing debate exists about the efficacy of combining CA with certain drugs. As the application of CA for cancer treatment is still in the animal or cellular experiments stage, further research is necessary to elucidate its efficacy and mechanism of action on different cancers.

## Author contributions

**Conceptualization:** Fangbing Ren, Yanhui Ma, Kexin Zhang, Fang Han, Xiaodong Sun.

**Data curation:** Fangbing Ren, Yanhui Ma, Kexin Zhang, Youhong Luo, Ruiyan Pan, Jingwen Zhang, Chengxia Kan, Ningning Hou.

**Investigation:** Youhong Luo, Ruiyan Pan, Jingwen Zhang, Chengxia Kan, Ningning Hou.

**Methodology:** Fangbing Ren, Yanhui Ma, Kexin Zhang.

**Supervision:** Fang Han, Xiaodong Sun.

**Writing – original draft:** Fangbing Ren, Yanhui Ma, Kexin Zhang.

**Writing – review & editing:** Fang Han, Xiaodong Xiaodong Sun.
